# Safety and Precision of Two Different Flap-morphologies Created During Low Energy Femtosecond Laser-assisted LASIK

**DOI:** 10.18502/jovr.v18i1.12720

**Published:** 2023-02-21

**Authors:** Johannes Steinberg, Juliane Mehlan, Bulat Mudarisov, Toam Katz, Andreas Frings, Vasyl Druchkiv, Stephan J Linke

**Affiliations:** ^1^Department of Ophthalmology, University Hospital Hamburg-Eppendorf, Hamburg, Germany; ^2^Hamburg Vision Clinic, Hamburg, Germany; ^3^CareVision GmbH, Hamburg, Germany; ^4^Univ.-Augenklinik Düsseldorf, Düsseldorf, Germany; ^5^Augenarztpraxis PD Dr. Frings, Nürnberg, Germany

**Keywords:** Cornea, Femtosecond Laser, Flap Morphology, LASIK, Refractive Surgery

## Abstract

**Purpose:**

Currently, two major principles exist to create LASIK flaps: firstly, a strictly horizontal (2D) cut similar to the microkeratome-cut and secondly an angled cut with a “step-like” edge (3D). The strictly horizontal (2D) cut method can be performed using apparatus such as the low-energy FEMTO LDV Z8 laser and its predecessors which are specific to this type. Alternatively, the low-energy FEMTO LDV Z8 laser's 3D flap design creates an interlocking flap-interface surface which potentially contributes toward flap stability. In addition, the FEMTO LDV Z8 offers flap-position adjustments after docking (before flap-creation). The current study analyzed precision, safety, efficacy, as well as patient self-reported pain and comfort levels after applying two different types of LASIK flap morphologies which were created with a low-energy, high-frequency femtosecond (fs) laser device.

**Methods:**

A prospective, interventional, randomized, contralateral eye, single-center comparison study was conducted from November 2019 to March 2020 at the Hamburg vision clinic/ zentrumsehstärke, Hamburg, Germany. Eleven patients and 22 eyes received low-energy fs LASIK treatment for myopia or myopic astigmatism in both eyes. Before the treatment, the eyes were randomized (one eye was treated with the 2D, the other eye with the 3D method).

**Results:**

The mean central flap thickness one month after surgery was 110.7 ± 1.6 μm (2D) and 111.2 ± 1.7 μm (3D); *P* = 0.365 (2D vs 3D). Flap thickness measured at 13 different points resulted in no statistically significant differences between any of the measurement points within/between both groups; demonstrating good planarity of the flap was achieved using both methods. Despite not being statistically significant, the surgeons recognized an increase in the presence of an opaque bubble layer in the 3D flap eyes during surgery and some patients reported higher, yet not statistically significant, pain scores in the 3D flap eyes during the first hours after the treatment. Overall, safety- and efficacy indices were 1.03 and 1.03, respectively.

**Conclusion:**

In this prospective, randomized, contralateral eye study, the low-energy fs laser yielded predictable lamellar flap thicknesses and geometry at one-month follow-up. Based on these results, efficacy and safety of the corresponding laser application, that is, 2D vs 3D, are equivalent.

##  INTRODUCTION

More than 30 years ago, laser in situ keratomileusis, better known as LASIK, started its journey to become the most frequently performed treatment to correct ametropia in otherwise healthy eyes. Over the past decades, several modifications were made to further improve its safety, efficacy, and predictability, as well as the comfort levels for both patients and surgeons.^[1,2]^ One of the improvements was to create a highly precise LASIK flap with a femtosecond (fs) laser to reduce variations in terms of flap thickness (FT) and flap-related complications when compared to microkeratome-created flaps.^[3,4]^

In addition, the fs laser enables different flap-morphology designs to potentially further improve the safety of the surgery. Currently, two methods exist to create LASIK flaps: firstly, a strictly horizontal two-dimensional (2D) flap-cutting geometry, comparable to that of the microkeratome and secondly, a three-dimensional (3D) flap-cutting geometry which is in essence a combination of the horizontal cut and an angled side-cut, leading to a “step-like” edge. The first option is only possible with the use of the low-energy FEMTO LDV Z8 laser (Ziemer Ophthalmic Systems AG, Switzerland) and its predecessors. Whilst the 3D method creates a perfectly fitting angled flap, the interface morphology is believed to contribute toward flap stability and might also lead to a decreased number of flap striae and/or epithelial ingrowth.^[5,6,7]^ However, due to the entrapped air emerging during the fs-laser “cutting” process of the horizontal flap-interface, the occurrence of opaque bubble layers (OBL) increases and potential tissue bridges might also occur in the 3D-flaps.^[8]^ Therefore, the LDV Z8 fs laser creates additional “venting tunnels” during the 3D flap preparation, which lead the gas formed during the flap-cutting process into the direction of the flap bed downward and outward, to allow the gas to dissipate out of the stroma. Several FT predictability (intended versus achieved) comparisons were made in the past, where multiple fs lasers were used and were responsible for creating the flaps.^[9,10,11]^

However, the current study was done to analyze the predictability, precision, safety, efficacy, as well as the patients' pain and comfort levels when comparing the application of two different flap morphologies which were both created using the same low-energy, high-frequency fs laser.

##  METHODS

This single-center (Hamburg Vision Clinic, Hamburg, Germany), prospective, interventional, randomized, contralateral eye study was conducted from November 2019 to March 2020. The study was registered in clinicaltrials.gov (NCT04426175) after Hamburg ethics committee approval and performed in accordance with the tenets of the Declaration of Helsinki. All patients signed an informed consent form after being advised in detail about the study rationale.

All patients received fs LASIK treatment for myopia or myopic astigmatism on both eyes from one of the two trained surgeons (SL/JS). The inclusion and exclusion criteria were similar to the criteria defined by the national committee defining the inclusion and exclusion criteria recommendations for refractive surgery in Germany (KRC). Hence, the inclusion criteria were: myopia up to 8 diopters, astigmatism up to 5 diopters, minimal corneal thickness of 480 μm, and a minimum of two weeks of no contact lens wearing. Patients with predicted residual stromal thickness (RST) under the flap after ablation of < 250 μm, former ocular surgery, ocular diseases (including, but not limited to, signs of keratoconus), aged younger than 18 years, and concurrent participation in another ophthalmological clinical study were excluded.

**Figure 1:**
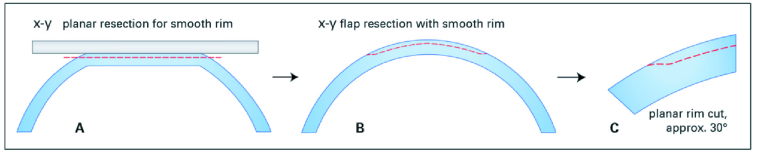
Flap morphology in planar (2D) flaps**. **Side view on the 2D LASIK flap. The flap is generated after applanation in a strictly horizontal plane leading to a minimum-angled flap edge after the applanation (A & B). Planar rim cut at approximately 30º (C). Source: Femto LDV Z8; Surgical procedure manual Cornea; Ziemer Ophthalmology.

**Figure 2:**
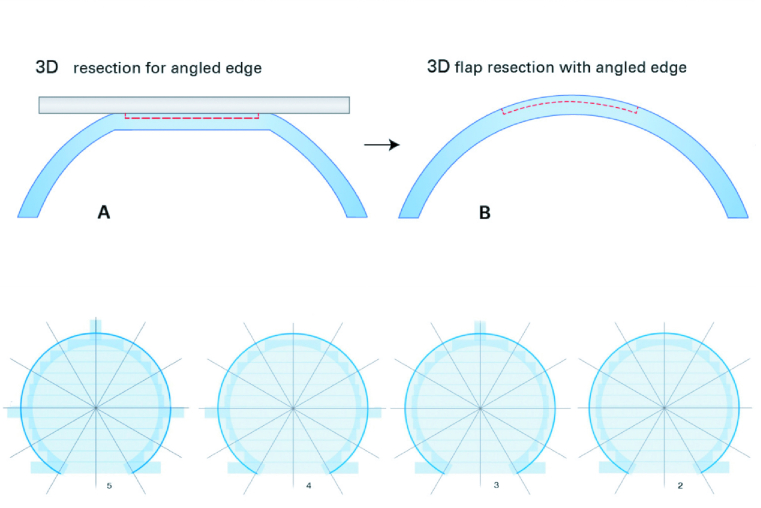
Flap morphology in planar (3D) flaps**. **Side (upper row) and top (lower row) views on the 3D LASIK flap. The flap is generated after applanation combining a strictly horizontal plane with a 90º side cut (A & B). Additional venting tunnels (optionally 2–5 tunnels, see lower row) have to be created to release the otherwise enclosed air. Source: Femto LDV Z8; Surgical procedure manual Cornea; Ziemer Ophthalmology.

Just before the treatment commenced, every patient was assigned according to our randomization list, those with a 2D flap created in one eye and those with a 3D-flap creation in the other eye. Schematic drawings of the morphology and geometry of both flap designs are displayed in Figures 1 (2D) and 2 (3D). In our study, the option of two venting tunnels (3D flap) was chosen [Figure 2].

In all eyes, the flap was created with the FEMTO LDV Z8 (Ziemer Ophthalmic Systems AG, Port) with a target thickness of 110 μm along with a superior hinge configuration. The subsequent excimer laser ablation was performed with the WaveLightⓇ EX500 Excimer Laser (Alcon Fort Worth, USA).

**Figure 3:**
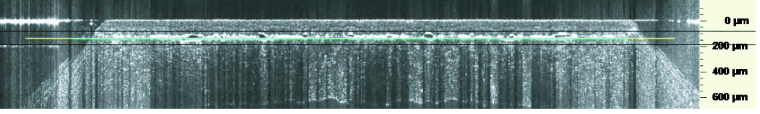
Intraoperative OCT before flap lifting. The yellow line defines the cut position.

An optical coherence tomography (OCT) image for flap visualization can be displayed, at the surgeon's discretion, on the screen before and/or after the flap resection. Before flap creation, flap visualization may serve as an optional safety measure confirming the flap's positioning with respect to the Bowman's layer and the stroma, or, post flap-creation, where the presence of gas bubbles can be assessed as an additional safety measure before flap lifting [Figure 3]. In addition, the intraoperative OCT feature can be useful for the visualization of the applanation area.

Antibiotic eyedrops were instilled for one week, while steroids and lubricants were reduced gradually over the course of one month.

The primary objective of this study was to compare central FT predictability in 110 µm LASIK flaps between 2D and 3D flap geometry groups with spectral domain anterior segments (AS)-OCT (Maestro 1, Topcon Medical Systems Tokyo, Japan) performed one month postoperatively. After measuring the cornea with the OCT using a scan protocol consisting of 12 B-scans in a radial pattern, the anterior surface was automatically marked in the image by the device. Three centrally located manual thickness measurements from the anterior surface to the interface were done and the average value was noted.

**Figure 4:**
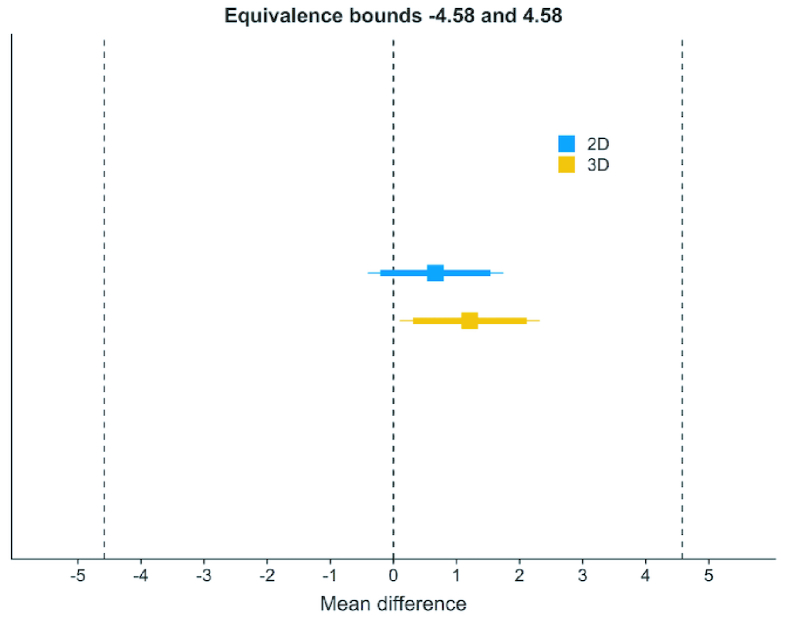
The mean differences of flap thickness from 110 µm. Squares: Mean differences from 110 µm. Dashed lines equivalence bounds (–4.58; 4.58). Thick line around differences is TOST (Two One-Sided Tests) confidence interval. 2D 90% CI (–0.207; 1.54); 3D 90% CI (0.311; 2.113). Thin line NHST (Null Hypothesis Significance Test) 95% confidence interval. 2D 95% CI (–0.407; 1.741); 3D 95% CI (0.105; 2.32).

Secondary objectives of the study were to assess the following parameters:

Postoperative flap planarity with AS-OCT (Maestro 1, Topcon, Japan) at one month follow-up. After measuring the cornea with the OCT using a scan protocol consisting of 12 B-scans in a radial pattern, the anterior surface was automatically marked in the image by the device. Then, three manual thickness measurements from the anterior surface to the interface were done in 12 different measurement points as displayed in Figure 4. For every measurement point, three consecutive measurements were done and the average was noted.

Subjective intraoperative flap morphology assessment included: Stromal bed quality, ease of flap lifting, and presence of OBL. The grading was given by the surgeon directly after the completion of the treatment and was based on their assessment during/after the flap lift.

Self-reported pain perception and visual experience with 2D and 3D flap geometries during the early postoperative period. During the one-day follow-up examination, patients were asked to respond to three different questions.

Safety and Efficacy index (EI) defined as the best-corrected visual acuity (BCVA) after treatment divided by corrected distance visual acuity (CDVA) before treatment (BCVA post/BCVA pre), and uncorrected visual acuity (UCVA) after treatment divided by BCVA before treatment (uncorrected distance acuity (UDVA) post/BCVA pre), respectively, were calculated.

### Statistical Analysis

Statistical analyses were performed using the SASⓇ software version 9.4 and R software (https://www.r-project.org). A two-sided *t*-test was used to test the primary hypothesis. Further two-sided *t*-tests were used to test the difference between the 2D and 3D flap geometries for continuous parameters, while Fisher's exact test was used to test categorical parameters. In the case of multiple comparisons (for instance, between the regions or distances) the *P*-values were adjusted with Holm's method.

The sample size was estimated using nQueryⓇ V4.0.

##  RESULTS

Eleven patients (22 eyes) were included in our study. Seven (63.6%) patients were female, while four (36.4%) were male. The mean age at the time of surgery was 37.27 ± 10.32 years (range: 21 to 54). Preoperative refraction and corneal thickness data are displayed in Table 1. None of the analyzed demographic, refractive or tomography parameters, displayed statistically significant differences between the 2D and 3D group (for all *P* > 0.05).

### Primary Objective

Postoperative central FT for the 2D and 3D cutting geometries were measured at the one-month follow-up visit. Target FT was 110 µm. The results are displayed in Table 2.

Figure 4 displays the differences from the target FT (110 µm). Based on the equivalence test and the null-hypothesis test combined, we can conclude that the observed effect is statistically not different from zero nor statistically equivalent to zero for 2D and 3D cutting geometries.

At the one-month follow-up visit, the mean central FT ± SD measured for 2D geometry flaps was 110.67 ± 1.60 μm, and for the 3D geometry flaps was 111.21 ± 1.65 μm. The mean difference ± SD between the target and achieved FT for each individual cutting geometry group was 0.67 ± 1.60 µm (2D) and 1.21 ± 1.65 µm (3D). Although the 2D cutting geometry group showed a lower mean difference in terms of FT predictability, the difference was not statistically significant (*P* = 0.440).

### Secondary Objectives

The results of the secondary objectives are displayed in Tables 3–5.

**Figure 5:**
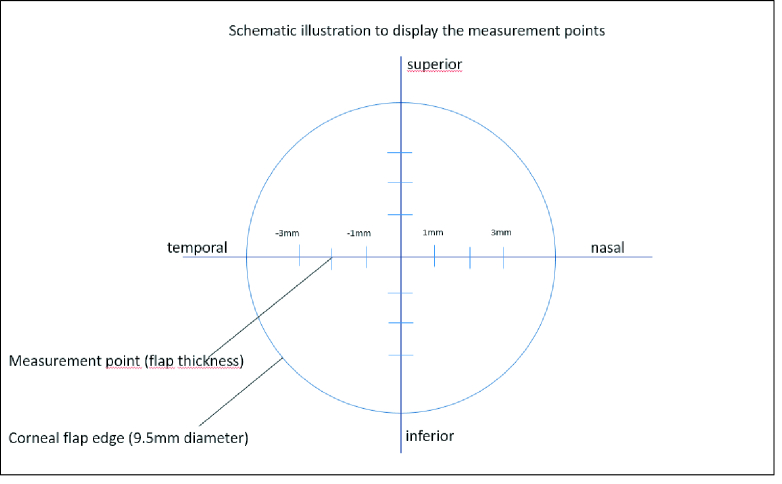
Schematic illustration to display the four measurement points in each meridian.

Concerning postoperative flap planarity at the one-month follow-up visit measured with AS-OCT; for both 2D and 3D flap cut geometry groups, a series of three consecutive thickness measurements were taken at four distinct measurement points ( ± 1, ± 2, and ± 3 mm from the center) along four different meridians (further called subgroups), namely: superior, inferior, nasal, and temporal [Figure 5]. The mean values were taken for each point and then the averages of the means were compared in terms of subgroups.

Data of the respective means and overall averages were analyzed [Table 3].

For our analyses, we allocated the measurement points to four subgroups: superior, inferior, nasal, and temporal. When comparing these subgroups, we couldn't demonstrate any statistical relevant differences among the four subgroups for either 3D or 2D flaps, neither when comparing the totals of the four subgroups of 3D with the totals of the four subgroups of 2D (all *P* > 0.05).

We also combined all horizontal and all vertical measurement points to analyze potential differences between the 2D and 3D flaps. Again, no statistically significant differences could be demonstrated (all *P* > 0.05). Furthermore, no statistically significant changes from the central to the periphery of the cornea (i.e., potentially increasing or decreasing flap thickness) could be demonstrated either for 2D or 3D flaps.

Regarding our secondary objective of analyzing subjective intraoperative flap morphology, we used a predefined grading and surgeon's assessment during and after the procedure where the flaps were lifted [Table 4]. Eight eyes (72.7%) in the 2D group which were compared to five eyes (45.5%) in the 3D group presented with a flat stromal bed (*P* = 0.387). Despite not being statistically significant, both surgeons found corneal stromal striae in two eyes (18.2%) (*P* = 0.476) and “rastered” interface in one eye (9.1%) (*P* = 1.000) in the 3D geometry group as compared to none in the 2D flap-cutting geometry group. Surgeons were able to easily lift nine flaps (81.8%) from the 2D and six flaps (54.5%) from the 3D group (*P* = 0.361). No OBL was found in nine eyes that received 2D flaps (81.8%) or six eyes that received 3D flaps (54.5%), respectively (*P* = 0.453).

Regarding the secondary objective of this study, analyzing postoperative subjective pain and visual perception, a questionnaire was completed during the first day follow-up visit where patients assessed their own visual quality and perceived pain levels during the first hours after the treatment.

Whereas more than half of the eyes treated with either a 2D or 3D flap morphology experienced none to moderate pain, reported during the first hours after surgery, higher pain scores were reported for some of the eyes treated with a 3D flap.

In summary, pain perception was slightly better in the 2D group, however, it was not statistically significant.

Regarding the visual assessment right after the treatment and at the beginning of the first day after surgery, no statistically or clinically significant differences could be demonstrated. Detailed results are displayed in Table 5.

The patients were also asked to give an assessment of their visual quality and pain level at the one-week follow-up visit. None of the patients reported any noticeable differences when comparing their 3D and 2D-flap eyes.

Table 6 provides a better representation of the functional parameters. Preoperative and postoperative UCVA data for the respective follow-up examinations are presented, and converted from Snellen notation to the logarithm of the minimum angle of resolution (logMAR).

The mean UCVA (*n* = 22) improved significantly (*P* < 0.0001) between the preoperative visit 0.97 ± 0.36 logMAR and the one-month follow-up visit 0.00 ± 0.02 logMAR.

**Table 1:** Descriptive summary (preoperative refraction and pachymetry) of the 2D and 3D groups.


	<@orange**2D (** ***n*** ** = 11)**			<@orange**3D (** ***n*** ** = 11)**		
orange**Parameter (unit)**	orange**Median (Q1/Q2)**	orange**Mean ± SD**	orange**Min/Max**	orange**Median (Q1/Q2)**	orange**Mean ± SD**	orange**Min/Max**
Sphere (D)	–2.00 (–3.25; –1.75)	–2.45 ** ± ** 1.36	–5.50/–1.0	–2.25 (–4.25; –1.50)	–2.61 ** ± ** 1.69	–6.25/–0.25
Cylinder (D)	–0.75 (–1.50; –0.50)	–0.93 ** ± ** 0.77	–2.75/0.00	–0.50 –1.00; –0.50)	–0.79 ** ± ** 0.69	–2.50/0.00
SE (D)	–2.38 (–4.00; –1.88)	–2.92 ** ± ** 1.43	–6.00/–1.50	–2.62 (–4.63; –1.75)	–3.01 ** ± ** 1.62	–6.25/–1.00
CCT (µm)	566 (531; 591)	564 ** ± ** 37	514/642	565 (533; 588)	564 ** ± ** 34	516/632
	
	
white<bcol>7</ecol>SE, spherical equivalent; CCT, central corneal thickness *P*-values from a two-sided *t*-test = all > 0.05

**Table 2:** Postoperative central flap thickness measured with AS-OCT at one-month follow-up visit.


	<@orange**2D**			<@orange**3D**			orange ***P*** **-value** ${}^{*}$
orange**Characteristics (unit)**	orange**Median (Q1/Q3)**	orange**Mean ± SD**	orange **Min/Max**	orange**Median (Q1/Q3)**	orange**Mean ± SD**	orange**Min/Max**	
Flap thickness achieved (µm)	110.3 (109.3/111.7)	110.67 ** ± ** 1.60	108.3/114.3	110.7 (110.3/111.3)	111.21 ** ± ** 1.65	109.3/114.3	0.440
Achieved thickness minus Target (µm)*	0.33 (–0.67/1.67)	0.67 ** ± ** 1.60	–1.7/4.3	0.67 (0.33/1.33)	1.21 ** ± ** 1.65	–0.7/4.3	0.440
	
	
white<bcol>8</ecol> ${}^{*}$ *P*-value from a two-sided *t*-test

**Table 3:** Mean flap thickness for 2D vs 3D flaps measured with AS-OCT from central along the superior, inferior, nasal, and temporal meridians at respective points: ± 1, ± 2, and ± 3 mm at one-month follow-up visit.


orange**2D** (*n* = 11)	orange**Central (µm) **Mean ** ± ** SD	orange ± **1 mm (µm) **Mean ** ± ** SD	orange ± **2 mm (µm) **Mean ** ± ** SD	orange ± **3 mm (µm) **Mean ** ± ** SD
Superior	110.67 ** ± ** 1.60	111.33 ** ± ** 1.56	111.85 ** ± ** 2.34	111.58 ** ± ** 2.80
Inferior	110.67 ** ± ** 1.60	111.61 ** ± ** 2.27	112.18 ** ± ** 1.82	111.76 ** ± ** 2.18
Nasal	110.67 ** ± ** 1.60	111.21 ** ± ** 2.30	110.91 ** ± ** 2.10	111.18 ** ± ** 2.72
Temporal	110.67 ** ± ** 1.60	111.12 ** ± ** 2.58	111.30 ** ± ** 1.64	110.70 ** ± ** 2.35
Overall average	**110.67 ± 1.60**	**111.32 ± 1.92**	**111.56 ± 1.69**	**111.30 ± 2.17**
**3D** (*n* = 11)	**Central (µm) **Mean ** ± ** SD	± **1 mm (µm) **Mean ** ± ** SD	± **2 mm (µm) **Mean ** ± ** SD	± **3 mm (µm) **Mean ** ± ** SD
Superior	111.21 ** ± ** 1.65	111.70 ** ± ** 1.74	111.85 ** ± ** 1.77)	111.58 ** ± ** 2.53
Inferior	111.21 ** ± ** 1.65	112.85 ** ± ** 1.98	111.73 ** ± ** 2.11	111.91 ** ± ** 2.23
Nasal	111.21 ** ± ** 1.65	111.33 ** ± ** 2.08	111.06 ** ± ** 2.63	112.52 ** ± ** 2.83
Temporal	111.21 ** ± ** 1.65	112.76 ** ± ** 3.30	111.33 ** ± ** 2.86	111.55 ** ± ** 2.37
Overall average	**111.21 ± 1.65**	**112.16 ± 1.85**	**111.49 ± 2.03**	**111.93 ± 2.24**
	
	

**Table 4:** Summary of intraoperative assessments: stromal bed quality, ease of flap lift, and presence of opaque bubble layer (OBL).


orange**Characteristics**	orange**Category**	orange**2D**	orange**3D**	orange ***P*** -value*
Stromal bed quality, *n* (%)	Smooth	8 (72.7)	5 (45.5)	0.387
	Tissue bridges	3 (27.3)	3 (27.3)	0.659
	Lines	0	2 (18.2)	0.476
	Rastered	0	1 (9.1)	1.000
		
Ease of flap lift, *n* (%)	Easily	9 (81.8)	6 (54.5)	0.361
	Sticky	2 (18.2)	5 (45.5)	
Presence of OBL, *n* (%)	No OBL	9 (81.8)	6 (54.5)	0.453
	< 30% of flap surface	1 (9.1)	2 (18.2)	
	30–40% of flap surface	1 (9.1)	3 (27.3)	
	
	
white<bcol>5</ecol>*P-value from a Fisher's exact test For stromal bed quality: because one eye could display more than one variable, *P*-values were given for each variable comparing 2D and 3D. In all other analyzes, the overall *P*-value for the Fisher's exact test was given.

**Table 5:** Summary of postoperative self-reported pain perception and visual experience stratified between 2D and 3D flap geometry groups on day-one follow-up visit.


orange**Category**	orange**2D(** ***n*** ** = 11)**	orange**3D(** ***n*** ** = 11)**	orange**Overall(** ***n*** ** = 22)**	orange ***P*** -value*
*“Rate your pain in the right eye/left eye during the hours after the treatment.”* No pain	4 (36.4)	3 (27.3)	7 (31.8)	0.821
Mild pain	2 (18.2)	1 (9.1)	3 (13.6)	
Moderate pain	3 (27.3)	2 (18.2)	5 (22.7)	
Severe pain	2 (18.2)	4 (36.4)	6 (27.3)	
Intense pain	0	1 (9.1)	1 (4.5)	
*Rate your first visual experience immediately after treatment:* *right eye/left eye.* As good as with glasses before treatment	2 (18.2)	2 (18.2)	4 (18.2)	1.000
Almost as good as with glasses before treatment	2 (18.2)	3 (27.3)	5 (22.7)	
A little blurred	4 (36.4)	3 (27.3)	7 (31.8)	
Blurry like seeing through foggy glasses	3 (27.3)	3 (27.3)	6 (27.3)	
“*Rate your visual acuity a few minutes after awakening this morning for the right and the left eye.” *As good as with glasses before treatment	5 (45.5)	5 (45.5)	10 (45.5)	1.000
Almost as good as with glasses before treatment	2 (18.2)	3 (27.3)	5 (22.7)	
A little blurred	4 (36.4)	3 (27.3)	7 (31.8)	
	
	
white<bcol>5</ecol> ${}^{*}$ P-value from a Fisher's exact test

**Table 6:** Summary of pre- and postoperative uncorrected visual acuity( logMAR).


orange**Characteristics**	orange**Category**	orange**2D(** ***n*** ** = 11)**	orange**3D(** ***n*** ** = 11)**	orange**Overall(** ***n*** ** = 22)**	orange ***P*** -value**
Preop UCVA	*n* (missing)	11 (0)	11 (0)	22 (0)	
	Mean (SD)	0.91 (0.32)	1.03 (0.39)	0.97 (0.36)	0.4224
	95% CI	0.69; 1.12	0.77; 1.30	0.81; 1.13	
	Median	1.00	1.00	1.00	
	Q1; Q3	0.70; 1.10	0.88; 1.10	0.80; 1.10	
	Min; Max	0.2; 1.3	0.4; 2.0	0.2; 2.0	
One day post-op UCVA	*n* (missing)	11 (0)	11 (0)	22 (0)	
	Mean (SD)	0.11 (0.08)	0.10 (0.04)	0.11 (0.06)	0.5668
	95% CI	0.06; 0.17	0.07; 0.13	0.08; 0.14	
	Median	0.10	0.10	0.10	
	Q1; Q3	0.10; 0.18	0.10; 0.10	0.10; 0.10	
	Min; Max	0.0; 0.3	0.0; 0.2	0.0; 0.3	
One week post-op UCVA	*n* (missing)	11 (0)	11 (0)	22 (0)	
	Mean (SD)	0.06 (0.08)	0.06 (0.11)	0.06 (0.09)	1.0000
	95% CI	0.01; 0.11	0.00; 0.14	0.02; 0.10	
	Median	0.00	0.00	0.00	
	Q1; Q3	0.00; 0.10	0.00; 0.18	0.00; 0.10	
	Min; Max	0.0; 0.2	0.0; 0.3	0.0; 0.3	
One month post-op UCVA	*n* (missing)	11 (0)	11 (0)	22 (0)	
	Mean (SD)	0.01 (0.03)	0.00 (0.00)	0.00 (0.02)	0.3293
	95% CI	0.00; 0.03	0.00; 0.00	0.00; 0.01	
	Median	0.00	0.00	0.00	
	Q1; Q3	0.00; 0.00	0.00; 0.00	0.00; 0.00	
	Min; Max	0.0; 0.1	0.0; 0.0	0.0; 0.1	
*P*-value*		< 0.0001	
	
	

### Safety and Efficacy

For all eyes combined, the overall mean UCVA (*n *= 22) measured at one month after treatment was 0.00 ± 0.02 logMAR. The safety index (SI) was 1.03, and the EI was 1.03 with no statistically significant differences between the 2D and 3D flap groups (all *P* > 0.05).

##  DISCUSSION

Published literature comparing microkeratome and fs laser created flaps is not a novelty anymore.^[3,4][13]^ Correspondingly, various publications exist comparing the predictability of FT measurements among fs lasers.^[9,11,14]^ To the best of our knowledge, this is the first study that compared the predictability of two flap-cutting geometries (2D vs 3D) created by the same low-energy high-spot density fs laser. In this prospective, randomized, contralateral, single-center study, we were able to demonstrate central FT predictability and discuss the objective and subjective intra- and postoperative flap morphology, as well as patients' visual and pain experience between the two groups. Furthermore, it was verified that both geometry groups displayed comparable overall linear and planar FTs from the center to the periphery of the cornea.

Concerning FT predictability in other studies, a retrospective series published in 2013 by Cummings et al where 120 µm intended thickness flaps were created by the FS200 fs laser (Alcon, Wavelight, Fort Worth, USA) in 162 eyes and measured by the AS-OCT (3D OCT-2000, Topcon Medical Systems Tokyo, Japan) postoperatively, showed a mean FT of 121.94 ± 10.52 µm.^[9]^

In another prospective study, 87 consecutive eyes received either 110 or 120 µm intended flaps created by the 200 kHz VisuMax fs laser (Carl Zeiss Meditec, Jena, Germany). Results showed a mean achieved FT of 112.3 ± 3.84 μm (range, 109.6 to 115.1) and 122.2 ± 3.93 μm (range, 115.8 to 129.0 μm) at one-month follow-up visit for the respective intended flaps created.^[15]^

In a study where 110 µm intended flaps were measured with FD-OCT one week postoperatively, results showed a mean central FT of 105.4 ± 3.4 µm for FS200 and 110.8 ± 3.9 µm for VisuMax-created flaps which was found to be statistically significant; *P* < 0.01.^[16]^ In the current study, although the mean central FT measured for 2D geometry flaps (110.67 ± 1.60 μm) was closer to the 110 µm target FT as compared to the 3D geometry flaps (111.21 ± 1.65 μm) at one-month follow-up, no statistically significant difference was found between the two groups; *P* = 0.440. Therefore, our results suggest that excellent predictability was achieved regardless of the flap geometry utilized, that is, 2D versus 3D.

One of the secondary objectives of our study was to analyze postoperative FT in both groups along 13 different data points across the horizontal and vertical meridians, starting from the center of the cornea to ± 1.0, ± 2.0, and ± 3.0 mm. Our results seem to be in line with the literature discussed below: Jagow et al demonstrated in their prospective comparative study, where FTs were created either by a 60 kHz fs laser (Intralase, Advanced Medical Optics) or a mechanical microkeratome (Zyoptix XP, Bausch & Lomb) and assessed with an AS-OCT for 20 points measured from the corneal vertex across each flap, for an intended 100 µm FT. The mean FT achieved ranged between 108 and 124 µm, with up to 16 µm of SD.^[17]^ Zheng et al compared flap morphology with the FD-OCT (uniformity, accuracy, predictability) of 110 µm intended flaps created by the FS200 (Alcon, Wavelight, Fort Worth, USA) (*n* = 200 eyes) and the VisuMax fs laser (Carl Zeiss Meditec, Jena, Germany) (*n* = 200 eyes), one week postoperatively. Nine thickness measurements were obtained across the length of the flaps at the meridians of 0º, 45º, 90º, and 135º with the cursor manually placed at ± 4, ± 3, ± 2, and ± 1 mm from the center of the flap. In total, 36 thickness measurements were analyzed for each flap. The mean FT achieved with the FS200 was 105.7 ± 2.6 µm, which was significantly less (*P* < 0.01) than for the VisuMax (111.2 ± 2.3 µm).^[17]^ Although both lasers used the 3D cutting geometry, the VisuMax results were found to be closer when corresponding to our 3D group. In our study, seeing that no statistically significant differences were found between the central and overall FT (13 points) results for both cutting geometry groups, we can conclude that both 2D and 3D flap-cutting geometries demonstrated high precision in terms of FTs.

Regarding our subjective intraoperative flap morphology findings, despite no statistically significant differences between both groups, both surgeons (SL, JS) noted from a clinical perspective a tendency toward a more homogeneous interface with a less adhesive flap and less OBL in the 2D flap geometry. Regarding postoperative self-reported pain perception and visual experience stratified between 2D and 3D flap geometry groups, the patients were unaware of which eye received 2D or 3D flap morphology (i.e., blinded study design). For seven eyes (31.8%) “no pain” was reported. Four of them belonged to the 2D group. However, despite not being statistically significant in our 22 eyes-analyses, higher pain scores were reported for some of the eyes treated with the “3D-flap”. Regarding the subjective visual quality assessment, no differences could be identified. Minutes after waking up the first morning post operation, for 10 eyes (equally distributed between 2D and 3D eyes), patients rated their visual acuity to be “as good as with glasses before treatment”. No statistically significant differences between intraoperative flap quality and patient's perception could be demonstrated during the first hours after the treatment. However, differences between both flap geometry groups regarding a tendency toward more adhesive and more OBL-prone flaps, potentially leading to the perceived (patient) discomfort in 3D flaps as compared to 2D flap geometry group, were clinically noticed.

Furthermore, it is important to keep in mind that the analyses of the intra-op flap-/interface morphology and the patients' postoperative assessments were secondary objectives of the study. Since the power analysis was based on the primary objective, that is, comparing the central FT predictability in both flap geometries and by taking the contralateral character of the study into account, we had to limit the number of eyes included in the study.

In case clinically relevant differences regarding subjective pain perception and morphology are to be assessed, bigger sample sizes would be necessary for future investigations. However, due to the low number of eyes included, the Fisher's exact test was used in these analyses. Our *P*-values > 0.05 indicate no correlation between the different categories and the 2D and 3D flap morphologies.

Technically, each flap-cutting geometry has its own special advantages. As mentioned, the 2D flap-cutting geometry only exists in the low-energy high-spot density FEMTO LDV Z8 laser and its predecessors.

Due to the nature of the 2D flap configuration, one would expect it to be relatively easy when lifting such a flap. With the FEMTO LDV Z8, not only are round flaps (as with the 2D) are a possibility but oval-shaped flaps can also be created with the 3D flap-cutting geometry tool. According to the patient requirements, 3D flap geometries can be resized or repositioned on the patient's eye, and side cut angles can be programmed from 30º to 150º around the globe.

The biggest advantage of the low-energy FEMTO LDV Z8 is the additional software option that exists called Optima which allows the surgeon to start with a 2D flap approach and switch intraoperatively to a 3D flap-mode after docking. As 2D flaps are generated without an angled site-cut, these flaps have to be created in the center of the applanated corneal surface. Adjusting the flap position after applanation would lead to a potentially irregular flap shape and/or a much too small or long hinge-configuration. Therefore, no such option for the surgeon exists other than in 3D flap-creation. With the 3D, angled side-cut geometry, even after applanation of the cornea, the surgeon can change settings including the flap position as well as the hinge position based on a live image. As mentioned before, the angled side cut in 3D geometry flaps creates a perfectly fitting angled flap and interface morphology believed to contribute toward flap stability which might lead to a decreased number of flap striae and/or epithelial ingrowth.^[5,6,7]^

Considering the aforementioned advantages and disadvantages of 2D versus 3D flap geometries as well as the flexibility to intraoperatively switch after docking in case the surgeon wants to shift the centration before flap-cutting commences, the low-energy FEMTO LDV Z8 seems to be the preferred choice in performing LASIK surgeries.

One of the limitations of our study was the lack of comparison of the induced corneal higher order aberrations (HOA) due to flap striaes which would be another important parameter when comparing the two flap-cutting techniques. As a result, it is recommended that we research this aspect for follow-up studies. Another limitation occurred with our small sample size as we had to limit the number of eyes included in our study to properly achieve our primary objective.

In summary, this study compared two different flap morphologies created during LASIK, whilst using the same low-energy, high-frequency OCT-equipped fs laser. Both the 2D and 3D flap-cutting geometries demonstrated comparable precision and predictability in terms of FTs combined with a high safety and efficacy performance.

### Financial Support and Sponsorship

None.

### Conflicts of Interest

None.
